# Mutation and expression analysis of the putative prostate tumour-suppressor gene PTEN.

**DOI:** 10.1038/bjc.1998.674

**Published:** 1998-11

**Authors:** I. C. Gray, L. M. Stewart, S. M. Phillips, J. A. Hamilton, N. E. Gray, G. J. Watson, N. K. Spurr, D. Snary

**Affiliations:** Imperial Cancer Research Technology, Applied Development Laboratory, St Bartholomew's Hospital, London, UK.

## Abstract

**Images:**


British Journal of Cancer (1998) 78(10), 1296–1300
© 1998 Cancer Research Campaign

### Mutation and expression analysis of the putative prostate tumour-suppressor gene *PTEN*

**IC Gray[1], LMD Stewart[1], SMA Phillips[2], JA Hamilton[1], NE Gray[1], GJ Watson[1], NK Spurr[3] and D Snary[1]**

[1]Imperial Cancer Research Technology, Applied Development Laboratory, Dominion House, St Bartholomew's Hospital, London EC1 7EB; [2]Department of Surgery, University of Birmingham, Birmingham B15 2TH; [3]Imperial Cancer Research Fund, Cancer Medicine Unit, St. James' University Hospital, Leeds LS9 7TF, UK

**Summary** The chromosomal region 10q23–24 is frequently deleted in a number of tumour types, including prostate adenocarcinoma and glioma. A candidate tumour-suppressor gene at 10q23.3, designated *PTEN* or *MMAC1*, with putative actin-binding and tyrosine phosphatase domains has recently been described. Mutations in *PTEN* have been identified in cell lines derived from gliomas, melanomas and prostate tumours and from a number of tumour specimens derived from glial, breast, endometrial and kidney tissue. Germline mutations in *PTEN* appear to be responsible for Cowden disease. We identified five *PTEN* mutations in 37 primary prostatic tumours analysed and found that 70% of tumours showed loss or alteration of at least one *PTEN* allele, supporting the evidence for *PTEN* involvement in prostate tumour progression. We raised antisera to a peptide from PTEN and showed that reactivity occurs in numerous small cytoplasmic organelles and that the protein is commonly expressed in a variety of cell types. Northern blot analysis revealed multiple RNA species; some arise as a result of alternative polyadenylation sites, but others may be due to alternative splicing.

**Keywords:** *PTEN*; prostate; cancer; mutation; expression

A number of chromosomal regions are frequently deleted in prostate tumours, suggesting that such loci harbour genes that suppress tumour development or progression. In particular, consistent losses at 8p, 16q and 10q have been observed (reviewed in Cannon-Albright and Eeles, 1995). Using fluorescence-based allelotyping, we previously identified a 9-cM interval at the 10q23/24 boundary that is deleted in most prostate tumours (Gray et al, 1995). Recently a candidate tumour-suppressor gene with putative actin-binding and tyrosine phosphatase domains has been identified at 10q23.3 and designated *PTEN* (Li et al, 1997) or *MMAC1* (Steck et al, 1997). Mutations in *PTEN* have been found in tumour specimens derived from glial, breast, endometrial and kidney tissue (Rhei et al, 1997; Steck et al, 1997; Tashiro et al, 1997; Wang et al, 1997) and in a number of melanoma (Guldberg et al, 1997) and prostate adenocarcinoma cell lines (Li et al, 1997; Steck et al, 1997). Germline mutations in *PTEN* have been identified in individuals with the autosomal dominant syndromes Cowden disease (multiple hamartoma syndrome) and Bannayan–Zonana syndrome. These disorders confer a predisposition to hamartomas at several sites, including the breast and thyroid (Liaw et al, 1997) and macrocephaly, lipomas, intestinal hamartomatous polyps and vascular malformations (Marsh et al, 1997).

Here we describe *PTEN* mutations in primary prostate tumours, supporting evidence that *PTEN* may act as a tumour suppressor in the prostate. In addition, we describe the generation of antipeptide antibody that detects PTEN in Western blots and localizes it within the cell. The complex *PTEN* mRNA expression profile is also analysed and discussed.

*Received 21 January 1998*

*Revised 21 April 1998*

*Accepted 22 April 1998*

*Correspondence to:* IC Gray, Smithkline Beecham Pharmaceuticals, Biopharmaceutical Research & Development, New Frontiers Science Park (North), Harlow, Essex CM19 5AW, UK

### MATERIALS AND METHODS

### Mutation analysis

Tumours and venous blood samples were obtained from men undergoing transurethral resection of the prostate. Tumour tissue was microdissected from normal tissue and tumour and blood DNA samples were prepared as described previously (Phillips et al, 1994). Using primers based on intron sequences, *PTEN* exons were amplified by polymerase chain reaction (PCR) from 30 ng of tumour DNA under the following conditions: an initial 95°C denaturing step of 2 min followed by 30 cycles of 95°C for 30 s, 60°C for 30 s and 72°C for 30 s in a 50-μl reaction volume. A 1-μl aliquot of product was then used to seed a second 15-cycle reaction using M13-21 tailed primers to facilitate dye-primer sequencing. After purification by passage through a Centricon-100 column (Amicon), exons were sequenced using a PRISM M13-21 dye-primer cycle sequencing system (Applied Biosystems). Mutations were confirmed by sequencing a second independently generated PCR product and comparing tumour-derived sequence with that obtained from matched blood DNA.

### Northern analysis

A multiple-tissue Northern blot (Clontech) was hybridized with a random-prime labelled gel purified insert from *PTEN* IMAGE consortium (Auffray et al, 1995) cDNA clone 264611 (Research Genetics) at high stringency in ExpressHyb hybridization solution (Clontech) in accordance with the supplier's instructions. A Northern blot consisting of mRNA from the lymphoblastoid cell line BRISTOL8 (BRI8; Snary et al, 1974) was hybridized with

**Table 1** *PTEN* mutations in prostate tumours

| Tumour stage[a] | Tumour grade[b] | 10q allele loss | Mutation | | Location |
|---|---|---|---|---|---|
| T4 M1 | 3 | Yes | insT: | normal: AGT-AAG<br>mutant: AGTTAAG | Exon 5 |
| T3 M1 | 3 | No | delG: | normal: TGGGATT<br>mutant: TGG-ATT | Exon 2 |
| T4 M1 | 2 | Yes | delTACT: | normal: TAGTACTTACTTT<br>mutant: TAG----TACTTT | Exon 8 |
| T2 M1 | 3 | Yes | Complex: | normal: GCAGAAAGACTTGAAG--GCGTATACA<br>mutant[c]: G**CAGAAAG**ACTTGAAGacagaaagACA | Exon 2 |
| T3 M0 | 3 | No | delT: | normal: GCTTCTCTTTTTTTTCTGTCCACCAG<br>mutant: GCTTCTCTTTTTTT-CTGTCCACCAG | Intron E (12 bp upstream of splice site) |

[a]Staging is based on digital rectal examination and bone scan. Four of the five tumours show metastasis (M1). [b]WHO gradings: 1, well differentiated; 2, moderately differentiated; 3, poorly differentiated; 4, mixture of differentiation. [c]The sequence in bold type appears to have duplicated and inserted downstream (lowercase type), giving an overall insertion of 2 bp.

individual PCR-amplified *PTEN* exons 1, 6 and 8, a 120-bp fragment derived from the *PTEN* 5′ untranslated region (UTR) and a 239-bp fragment from the 3′-UTR beyond the first polyadenylation signal, all at low stringency. Primer sequences for amplification of the 5′-UTR fragment were 5′-GGTCTGAGTCGCCTGTCACC-3′ and 5′-TTAAAACCGGCCCGGGTCCC-3′; primers for amplification of the 3′-UTR fragment were 5′-GACATTCGAGGAATTG-GCCGC-3′ and 5′-CAAGCCCATTCTTGTTGATAGCC-3′. PCR was performed under the conditions described above. Probes were generated by subsequent reamplification of 5 ng of PCR product for 11 rounds of 94°C for 1 min, 50°C for 1 min and 72°C for 1 min in the presence of 30 μCi of [α-$^{32}$P]dCTP (Hirst et al, 1992).

### Polyclonal antibody preparation, Western blotting and immunofluorescence

A peptide of 19 residues from amino acid positions 342 to 360 (sequence KVKLYFTKTVEEPSNPEAS) was synthesized and conjugated to keyhole limpet haemocyanin (KLH) using glutaraldehyde, 2 mg of peptide to 2 mg of protein (Coligan et al, 1994). Rabbits were immunized with the conjugate, 100 μg per immunization, in complete Freund's adjuvant on the first occasion and then on five subsequent occasions in incomplete Freund's adjuvant; the immunizations were at 2-week intervals. Antibody levels were determined by enzyme-linked immunosorbent assay (ELISA) using peptide conjugated to bovine serum albumin (BSA) and the specificity confirmed by Western blotting on bacterially expressed PTEN. Western blots were carried out on total cell lysates dissolved in SDS-PAGE sample buffer. Proteins were visualized using an alkaline phosphatase-based chemiluminescence system (Tropix, Applied Biosystems) and sized using BioRad low-molecular-weight standards. Immunfluorescence was performed with antibody that had been purified by protein A chromatography to reduce the non-specific cell-surface fluorescence found in the serum prior to immunization. FITC-conjugated sheep anti-rabbit IgG was used as a second antibody and immunofluorescent staining was visualized using a confocal microscope.

### RESULTS

Thirty-seven primary prostate tumours, of which 24 had previously shown allele loss at 10q23.3 (Gray et al, 1995; ICG unpublished data), were assessed for mutations in all nine *PTEN* exons (Steck et al, 1997) by direct sequencing following PCR amplification. Five mutations were identified (Table 1), four of which result in a truncated protein, supporting the hypothesis that *PTEN* is a prostate tumour-suppressor gene. Four of the mutations cause frameshifts (two deletions, one insertion and a complex combined deletion/duplication event). The remaining mutation is a small intronic deletion close to an intron/exon junction and which may cause aberrant splicing by reducing the length of the splice acceptor polypyrimidine tract (Shapiro and Senapathy, 1987). None of these mutation events was detected in matched blood samples and they therefore must have arisen somatically in the tumour.

Four intronic variants were also detected, each being present in both tumour and blood DNA: a single-base A→G substitution in intron A 96 bp upstream of exon 2; a 4-bp TTTG deletion in intron B 23 bp upstream of exon 3; a 5-bp ATCTT insertion in intron D 110 bp downstream of exon 4, and a T insertion also in intron D 28 bp upstream of exon 5. The allele with the 5-bp insert was found at a frequency of 39/74 in the 37 individuals studied. The remaining variants were less common, each being identified only once.

When used to probe a multiple-tissue Northern blot (Clontech), *PTEN* cDNA clone 264611 (Auffray et al, 1995), comprising exons 1–7 plus 478 bp of 5′ untranslated DNA (IC Gray, unpublished data), hybridizes to at least five bands common to all tissues tested with varying relative band intensities in each tissue type (Figure 1a). However, when a 120-bp fragment from the *PTEN* 5′-UTR was used to probe a similar blot, a single band of approximately 5.5 kb was identified in all tissues (Figure 1b). To determine further the relationship between the different transcripts, mRNA from the lymphoblastoid cell line BRI8 (Snary et al, 1974) was hybridized with probes derived from the *PTEN* 5′-UTR and with individual *PTEN* coding exons 1, 6 and 8. The 5′-UTR probe identified the expected 5.5-kb band observed with the multiple-tissue Northern blot (Figure 1ci). Probes derived from *PTEN* exons 1, 6 and 8 each gave a similar profile to the longer cDNA probe, with a 2.4-kb band consistently generating the strongest signal (Figure 1cii).

Examination of overlapping *PTEN* expressed sequence tags in the GenBank database suggests that a less intense 2.7-kb band (Figure 1a) represents an extension of the 2.4-kb species, the former having 300 bp of extra 3′ untranslated sequence owing to the use of an alternative polyadenylation signal. Consistent with

© Cancer Research Campaign 1998

**Figure 1** *PTEN* expression profile in a range of human tissues. (**A**) Labelled IMAGE cDNA clone 264611 (Auffray et al, 1995), consisting of *PTEN* exons 1–7 plus 478 bp of 5′ untranslated DNA (IC Gray, unpublished data), gives a similar expression pattern of at least five transcripts ranging from 2.4 to 5.5 kb in all tissues. Relative band intensities appear to vary between tissues. (**B**) A probe derived from the *PTEN* 5′ untranslated region detects a single 5.5-kb transcript in all tissues tested. (**C**) *PTEN* expression in the lymphoblastoid cell line BR18. Arrows show the migration positions of 18 and 28S ribosomal RNA. (i) The 5′-UTR probe detects the expected 5.5-kb transcript on a blot of BRI8 mRNA. (ii) A probe derived from *PTEN* exon 6 gives a similar profile to that seen with the longer cDNA probe in (**A**) above, with the 2.4-kb band giving the strongest signal. Identical patterns were produced with exons 1 and 8 (not shown). (iii) A probe from the *PTEN* 3′-UTR downstream of the first polyadenylation signal does not hybridize to the 2.4-kb transcript but detects the others, suggesting that the 2.4-kb mRNA uses this proximal polyadenylation site, whereas the others do not

**Figure 2** Western blot of a number of cell lines probed with rabbit antiserum to the C-terminal peptide of PTEN. POC+15 is an adenocarcinoma line from colon, MOLT4 a T-lymphoblastoid line, HACAT an epithelial cell line, MKN45 a gastric' carcinoma cell line, BRISTOL8 a B-lymphoblastoid cell line and HCT116 a colorectal carcinoma cell line. The predicted size of the PTEN protein (48.2 kDa) is indicated with an arrow

**Figure 3** Immunofluorescence staining of the DU145 prostate carcinoma cell line with anti-PTEN peptide rabbit antiserum. (**A**) Cells labelled with anti-PTEN antibody and (**B**) cell labelled with normal rabbit antibody. The figure in the top corner represents the distance from the slide that the photograph was taken this, a probe derived from this extra 3′ sequence does not hybridize to the 2.4-kb transcript, but detects the others from 2.7 to 5.5 kb (Figure 1ciii).

Two peptides were used to immunize rabbits, but only the most C-terminal of the two (amino acids 342–360) produced an antibody response. The other peptide, a 22-amino-acid sequence from position 219 to 240, did not induce an anti-PTEN response, although a good anti-carrier (anti-KLH) response was given. On Western blots the antiserum to the C-terminal peptide bound to a protein with an apparent molecular weight of 54.8 kDa, close to the predicted molecular weight from the PTEN amino acid sequence (48.2 kDa). Several cell lines gave an identical pattern (Figure 2). Immunofluorescence of permeabilized cells suggested that the antipeptide antibody binds to a small particulate structure within the cytoplasm of the cell (Figure 3).

### DISCUSSION

Although the identification of *PTEN* mutations in primary prostate tumours provides good evidence that *PTEN* is a prostate tumour-suppressor gene, the number of mutations detected is far lower than expected (5/37) given that nearly 70% of prostate tumours show loss of the 10q23.3 region (Gray et al, 1995; IC Gray, unpublished data). There are several possible explanations. Sequencing as a method of mutation detection is unlikely to be 100% efficient. The

 © Cancer Research Campaign 1998

nature of prostate tumour growth with a lack of normal tumour boundary makes it difficult to be certain about the level of normal tissue contamination of the dissected tumour. Furthermore, where functional loss is associated with the later stages of tumour progression, as appears to be the case here, there may be clonal subpopulations of tumour that do not carry the mutation. In addition, gross deletions spanning one or more exons and mutations in regulatory sequences outside the coding region would have gone undetected.

Alternatively, there is the possibility that mutation or loss of a single *PTEN* allele may be sufficient for a tumour growth advantage; our analysis showed three tumours with loss of one *PTEN* allele and a mutation in the second, 21 with loss of one *PTEN* allele but no detectable mutation in the second and two with one mutant allele but no detectable loss of the second. In summary, 26 tumours of a total of 37 (70%) had alteration or loss of at least one copy of *PTEN*. During preparation of this manuscript, a report appeared in the literature describing inactivation of both *PTEN* alleles in 10 of 80 primary prostate tumours studied (Cairns et al, 1997), providing further evidence that *PTEN* is a prostate tumour suppressor gene. Furthermore, prostate cancer has been identified in association with Cowden disease (Inagaki and Ebisuno, 1996), which has recently been shown to be caused by germline *PTEN* mutations (Liaw et al, 1997). However, the possibility of a further tumour-suppressor gene at 10q23.3 cannot be excluded.

*PTEN* appears to be expressed in a wide range of cell types; this is evident from the ubiquitous expression of the mRNA in all tissues examined and from the presence of the protein in cells from several different origins. The anti-PTEN antibody indicates that PTEN is found associated with small cytoplasmic particles, an observation in keeping with data describing the direct visualization of expressed PTEN protein with the Flag epitope (Li and Sun, 1997).

A complex pattern of transcripts was found for *PTEN* in all tissues tested, similar to the profiles previously reported by Steck et al (1997). Although some of the transcript profile may be accounted for by alternative polyadenylation sites, other differences are also evident, suggesting alternative *PTEN* splicing or cross-hybridization of *PTEN* with mRNA species of distinct but related sequence, raising the possibility that *PTEN* may be a member of a wider gene family. There appear to be at least two discrete major *PTEN* transcripts with 5′ sequence differences. Recently, an expressed *PTEN* pseudogene on chromosome 9 has been identified (Kim et al, 1998; Teng et al, 1998). Cross-hybridization to RNA derived from this pseudogene may therefore account for some of the *PTEN* transcript profile.

The broad spectrum of tumour types showing *PTEN* mutations (Li et al, 1997; Steck et al, 1997), coupled with apparently ubiquitous expression, suggests that *PTEN* has a role in the progression of a significant proportion of tumours derived from a diverse range of tissues. The identification of germline mutations in individuals suffering from Cowden disease (Liaw et al, 1997) raises the possibility that low-penetrance germline *PTEN* lesions may be responsible for some breast (and other) cancers previously thought to be sporadic. As four of the five mutations described here were detected in late-stage tumours showing metastasis (Table 1), *PTEN* inactivation may be involved in a pathway leading to metastatic potential; a recent analysis of metastatic prostate cancer tissues also implicates *PTEN* involvement in metastasis (Suzuki et al, 1998). Therefore, it could prove to be a useful marker for monitoring prostate tumour progression and provide information that will assist in making therapeutic decisions.

### ACKNOWLEDGEMENTS

This work was supported by the Imperial Cancer Research Fund and Zeneca Diagnostics.

### REFERENCES

Auffray C, Behar G, Bois F, Bouchier C, Da Silva C, Devignes MD, Duprat S, Houlgatte R, Jumeau MN, Lamy B, Lorenzo F, Mitchell H, Mariagesamson R, Pietu G, Pouliot Y, Sebastianikabaktchi C and Tessier A (1995) IMAGE: molecular integration of the analysis of the human genome and its expression. *C R Acad Sci III* **318**: 263–272

Cairns P, Okami K, Halachmi S, Halachmi N, Esteller M, Herman JG, Jen J, Isaacs WB, Bova GS and Sidransky D (1997) Frequent inactivation of PTEN/MMAC1 in primary prostate cancer. *Cancer Res* **57**: 4997–5000

Cannon-Albright L and Eeles R (1995) Progress in prostate cancer. *Nature Genet* **9**: 336–338

Coligan JE, Kruisbecek AM, Margulies DH, Shevach EM and Strober W (eds) (1994) *Current Protocols in Immunology*. Greene Publishing Associates and John Wiley: New York

Inagaki T and Ebisuno S (1996) A case of Cowden's disease accompanied by prostatic cancer. *Br J Urol* **77**: 918–919

Gray IC, Phillips SMA, Lee SJ, Neoptolemos JP, Weissenbach J and Spurr NK (1995) Loss of the chromosomal region 10q23-25 in prostate cancer. *Cancer Res* **55**: 4800–4803

Guldberg P, Straten PT, Birck A, Ahrenkiel V, Kirkin AF and Zeuthen J (1997) Disruption of the MMAC1/PTEN gene by deletion or mutation is a frequent event in malignant melanoma. *Cancer Res* **57**: 3660–3663

Hirst MC, Bassett JH, Roche A and Davies KE (1992) Preparation of radiolabelled hybridisation probes by STS labelling. *Trends Genet* **8**: 6–7

Kim SK, Su LK, Oh Y, Kemp BL, Hong WK and Mao L (1998) Alterations of PTEN/MMAC1, a candidate tumor suppressor gene, and its homologue, PTH2, in small cell lung cancer cell lines. *Oncogene* **16**: 89–93

Li DM and Sun H (1997) TEP1, encoded by a candidate tumour suppressor locus, is a novel protein tyrosine phosphatase regulated by transforming growth factor β. *Cancer Res* **57**: 2124–2129

Li J, Yen C, Liaw D, Podsypanina K, Bose S, Wang SI, Puc J, Miliaresis C, Rodgers L, McCombie R, Bigner SH, Giovanella BC, Ittmann M, Tycko B, Hibshoosh H, Wigler MH and Parsons R (1997) PTEN, a putative protein tyrosine phosphatase gene mutated in human brain, breast and prostate cancer. *Science* **275**: 1943–1947

Liaw D, Marsh DJ, Li J, Dahlia PLM, Wang SI, Zheng Z, Bose S, Call KM, Tsou HC, Peacocke M, Eng C and Parsons R (1997) Germline mutations of the PTEN gene in Cowden disease, an inherited breast and thyroid cancer syndrome. *Nature Genet* **16**: 64–67

Marsh DJ, Dahlia PLM, Zheng Z, Liaw D, Parsons R, Gorlin R and Eng C (1997) Germline mutations in PTEN are present in Bannayan-Zonana syndrome. *Nature Genet* **16**: 333–334

Phillips SMA, Morton DG, Lee SJ, Wallace DMA and Neoptolemos JP (1994) Loss of heterozygosity of the retinoblastoma and adenomatous polyposis susceptibility gene loci and in chromosomes 10p, 10q and 16q in human prostate cancer. *Br J Urol* **73**: 390–395

Rhei E, Kang L, Bogomolniy F, Federici MG, Bogan PI and Boyd J (1997) Mutation analysis of the putative tumor suppressor gene PTEN/MMAC1 in primary breast carcinomas. *Cancer Res* **57**: 3657–3659

Shapiro MB and Senapathy P (1987) RNA splice junctions of different classes of eukaryotes: sequence statistics and functional implications in gene expression. *Nucleic Acids Res* **15**: 7155–7174

Snary D, Goodfellow P, Hayman MJ, Bodmer WF and Crumpton MJ (1974) Subcellular separation and molecular nature of human histocompatibility antigens (HL-A). *Nature* **247**: 457–461

Steck PA, Pershouse MA, Jasser SA, Jung WK, Lin H, Ligon AH, Langford LA, Baumgard ML, Hattier T, Davis T, Frye C, Hu R, Swedlund B, Teng DHF and Tavtigian SV (1997) Identification of a candidate tumour suppressor gene, MMAC1, at chromosome 10q23.3 that is mutated in multiple advanced cancers. *Nature Genet* **16**: 64–67

Suzuki H, Freije D, Nusskern DR, Okami K, Cairns P, Sidransky D, Isaacs WB and Bova GS (1998) Interfocal heterogeneity of PTEN/MMAC1 gene alterations in multiple metastatic prostate cancer tissues. *Cancer Res* **58**: 204–209

Tashiro H, Blazes MS, Wu R, Cho KR, Bose S, Wang SI, Li J, Parsons R and Ellenson LH (1997) Mutations in PTEN are frequent in endometrial carcinoma but rare in other common gynecological malignancies. *Cancer Res* **57**: 3935–3940

© Cancer Research Campaign 1998 

Teng DH, Hu R, Lin H, Davis T, Iliev D, Frye C, Swedlund B, Hansen KL, Vinson VL, Gumpper KL, Ellis L, El-Naggar A, Frazier M, Jasser S, Langford LA, Lee J, Mills GB, Pershouse MA, Pollack RE, Tornos C, Troncoso P, Yung WK, Fujii G, Berson A, Bookstein R, Bolen JB, Tavtigian SV and Steck PA (1998) MMAC1/PTEN mutations in primary tumor specimens and tumor cell lines. *Cancer Res* **57**: 5221–5225

Wang SI, Puc J, Li J, Bruce JN, Cairns P, Sidransky D and Parsons R (1997) Somatic mutations of PTEN in glioblastoma multiforme. *Cancer Res* **57**: 4183–4186

© Cancer Research Campaign 1998


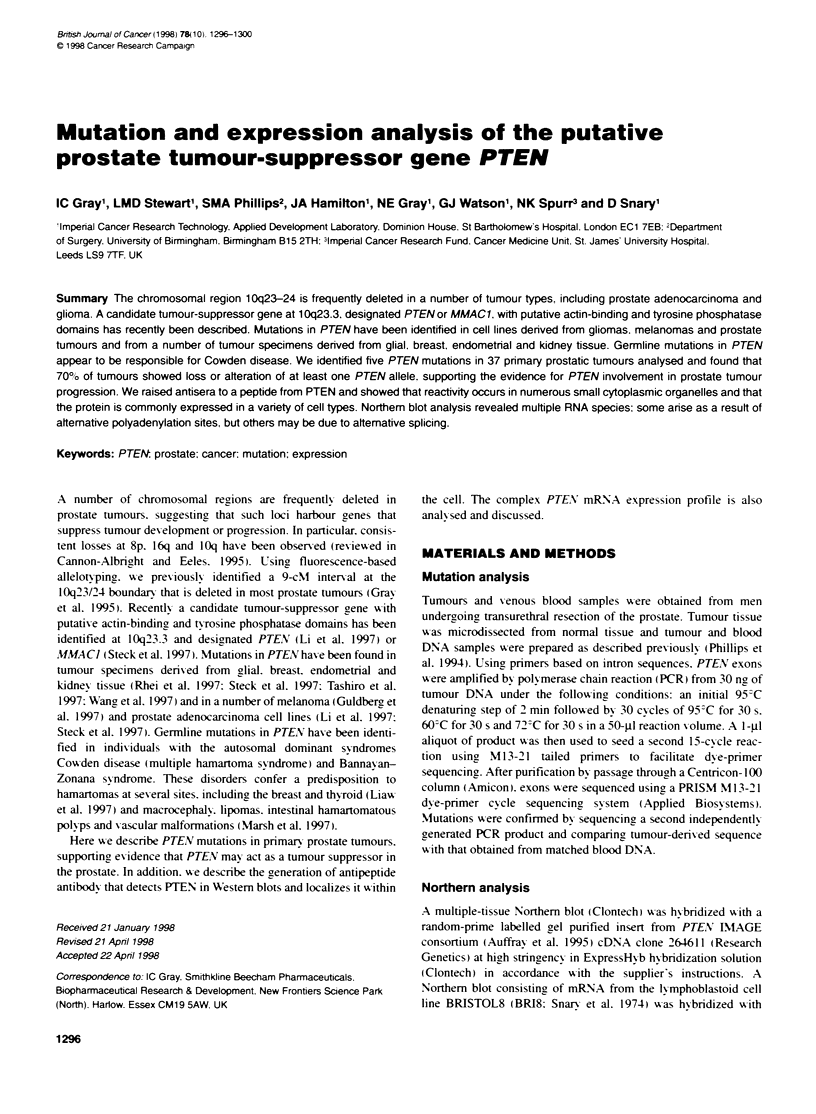

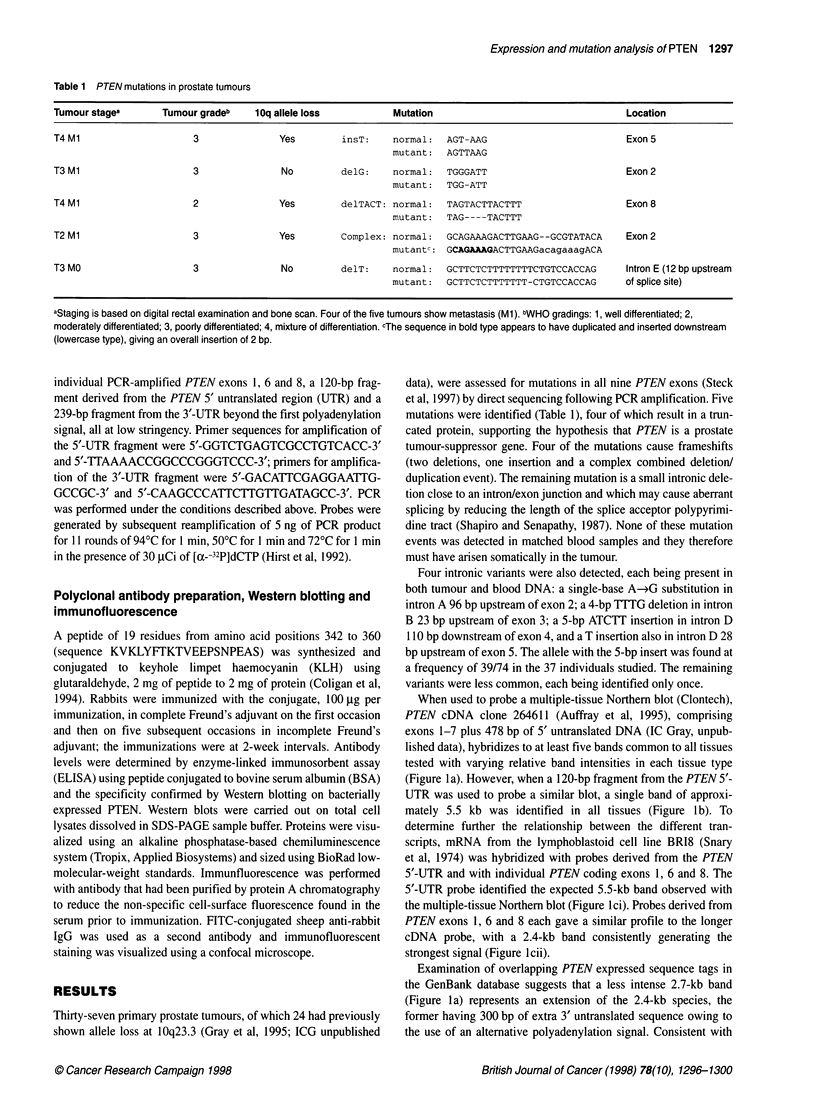

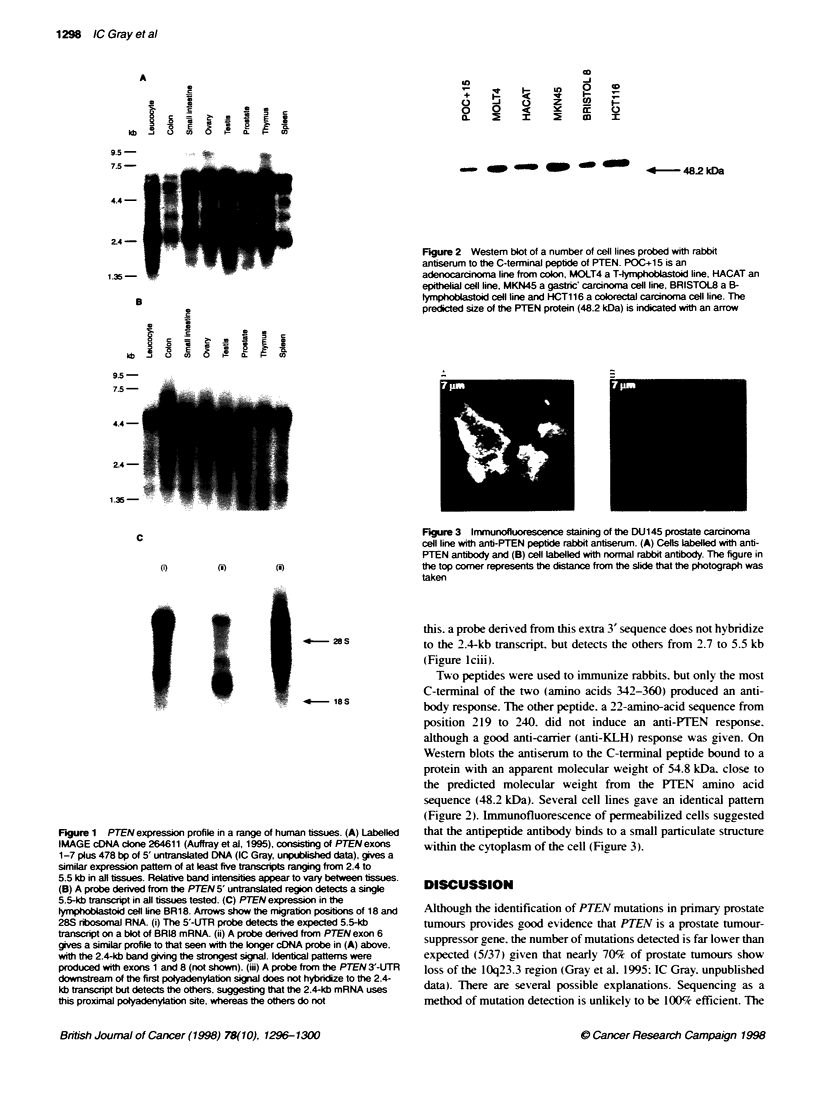

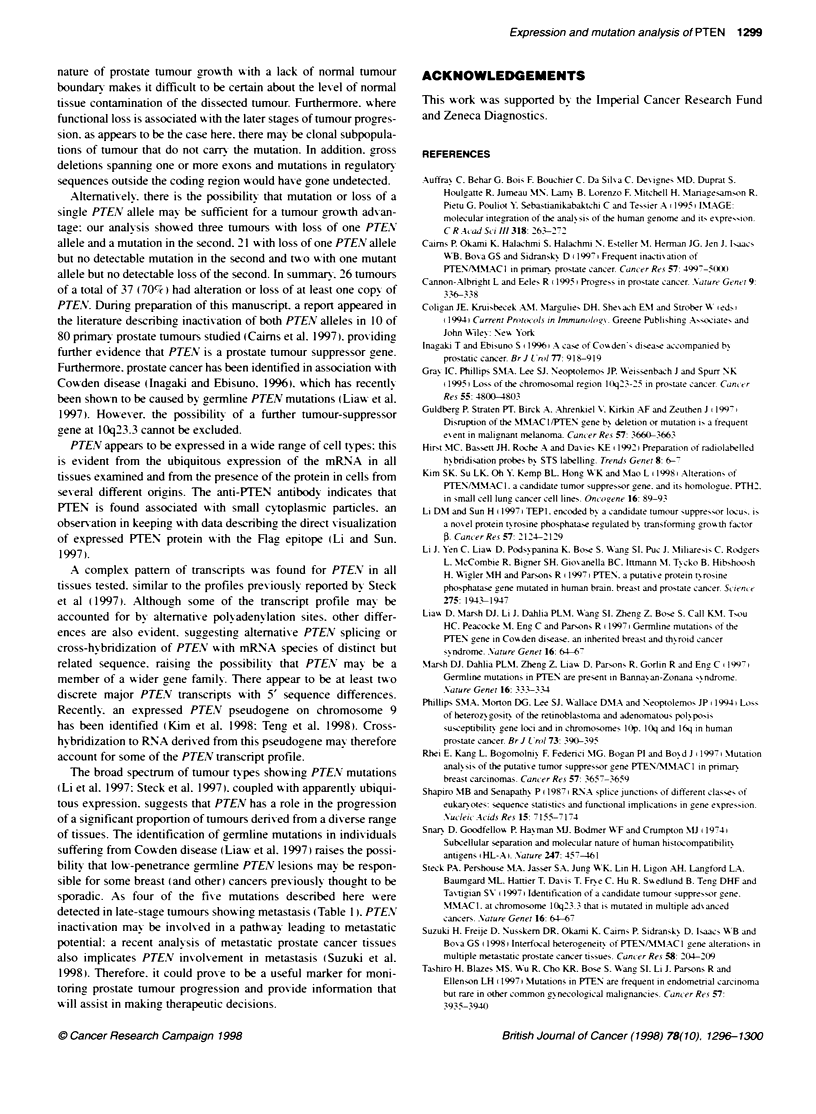

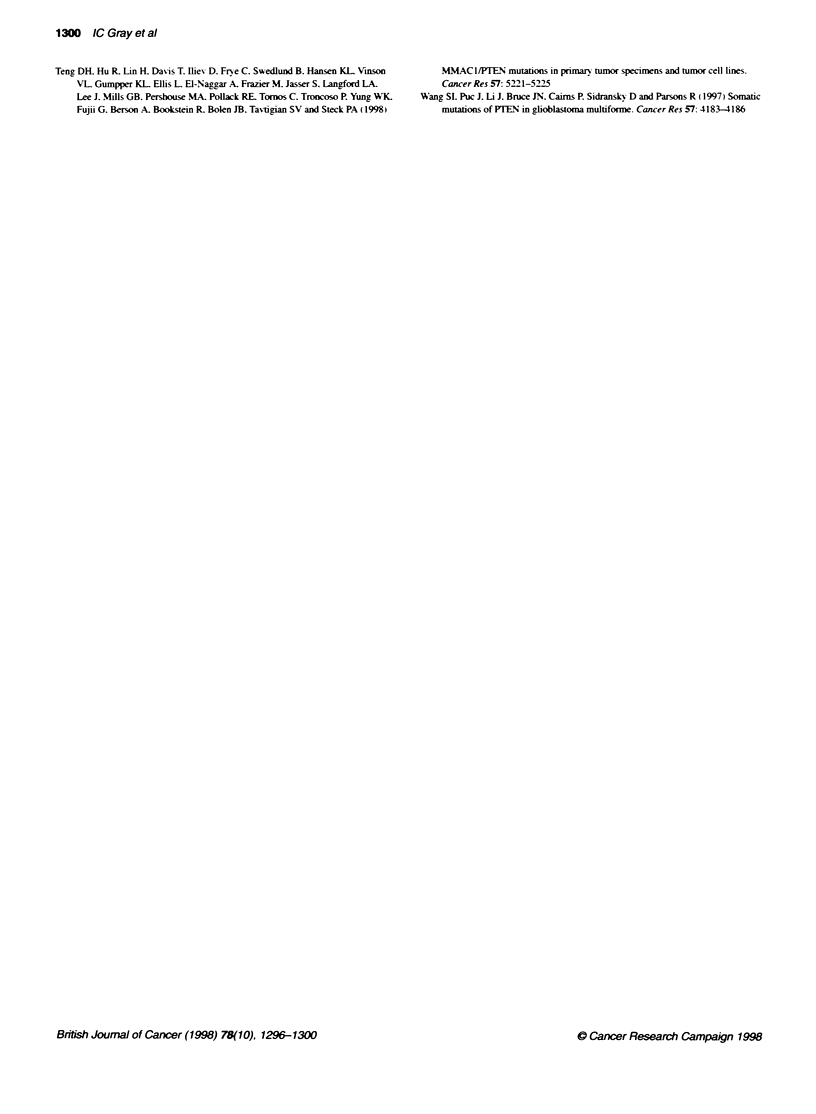


## References

[OCR_00439] Cairns P., Okami K., Halachmi S., Halachmi N., Esteller M., Herman J. G., Jen J., Isaacs W. B., Bova G. S., Sidransky D. (1997). Frequent inactivation of PTEN/MMAC1 in primary prostate cancer.. Cancer Res.

[OCR_00470] Kim S. K., Su L. K., Oh Y., Kemp B. L., Hong W. K., Mao L. (1998). Alterations of PTEN/MMAC1, a candidate tumor suppressor gene, and its homologue, PTH2, in small cell lung cancer cell lines.. Oncogene.

[OCR_00475] Li D. M., Sun H. (1997). TEP1, encoded by a candidate tumor suppressor locus, is a novel protein tyrosine phosphatase regulated by transforming growth factor beta.. Cancer Res.

[OCR_00478] Li J., Yen C., Liaw D., Podsypanina K., Bose S., Wang S. I., Puc J., Miliaresis C., Rodgers L., McCombie R. (1997). PTEN, a putative protein tyrosine phosphatase gene mutated in human brain, breast, and prostate cancer.. Science.

[OCR_00488] Marsh D. J., Dahia P. L., Zheng Z., Liaw D., Parsons R., Gorlin R. J., Eng C. (1997). Germline mutations in PTEN are present in Bannayan-Zonana syndrome.. Nat Genet.

[OCR_00506] Rhei E., Kang L., Bogomolniy F., Federici M. G., Borgen P. I., Boyd J. (1997). Mutation analysis of the putative tumor suppressor gene PTEN/MMAC1 in primary breast carcinomas.. Cancer Res.

[OCR_00516] Snary D., Goodfellow P., Hayman M. J., Bodmer W. F., Crumpton M. J. (1974). Subcellular separation and molecular nature of human histocompatibility antigens (HL-A).. Nature.

[OCR_00529] Suzuki H., Freije D., Nusskern D. R., Okami K., Cairns P., Sidransky D., Isaacs W. B., Bova G. S. (1998). Interfocal heterogeneity of PTEN/MMAC1 gene alterations in multiple metastatic prostate cancer tissues.. Cancer Res.

[OCR_00534] Tashiro H., Blazes M. S., Wu R., Cho K. R., Bose S., Wang S. I., Li J., Parsons R., Ellenson L. H. (1997). Mutations in PTEN are frequent in endometrial carcinoma but rare in other common gynecological malignancies.. Cancer Res.

[OCR_00541] Teng D. H., Hu R., Lin H., Davis T., Iliev D., Frye C., Swedlund B., Hansen K. L., Vinson V. L., Gumpper K. L. (1997). MMAC1/PTEN mutations in primary tumor specimens and tumor cell lines.. Cancer Res.

[OCR_00551] Wang S. I., Puc J., Li J., Bruce J. N., Cairns P., Sidransky D., Parsons R. (1997). Somatic mutations of PTEN in glioblastoma multiforme.. Cancer Res.

